# P-1247. Cerebrospinal Fluid Penetration of Multiple Antibiotics in Patients With Intracranial Hemorrhage: A Prospective, Observational, Clinical Study

**DOI:** 10.1093/ofid/ofae631.1429

**Published:** 2025-01-29

**Authors:** Yu-Ju Tseng, Yi-hsuan Chen, Yi-Xuan Lan, Chien-Chih Wu, Hui-Tzung Luh

**Affiliations:** National Taiwan University Hospital, Taipei, Taipei, Taiwan (Republic of China); National Taiwan University, Taipei, Taipei, Taiwan; National Taiwan University Hospital, Taipei, Taipei, Taiwan (Republic of China); National Taiwan University (NTU) Hospital, Taipei, Taipei, Taiwan; National Taiwan University Hospital, Taipei, Taipei, Taiwan (Republic of China)

## Abstract

**Background:**

External ventricular drains (EVD) are used for acute hydrocephalus in intracranial hemorrhage(ICH), potentially causing ventriculostomy-associated cerebrospinal fluid infection (VAI). Variable meningeal inflammation in VAI can result in unpredictable antibiotic penetration in cerebrospinal fluid (CSF). This observational study aimed to assess CSF penetration of intravenous antibiotics for suspected VAI treatment in neurosurgical patients.

Time to concentrations in CSF and plasma among the patients.

**Figure:**
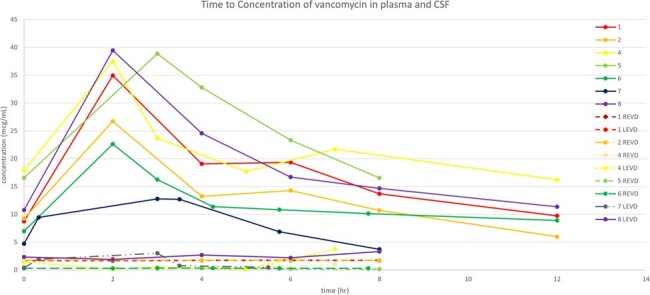

The figure presented the concentrations of blood and cerebrospinal fluid (CSF) measured at different time points for each patient. REVD right-sided external ventricular drain. LEVD left-sided external ventricular drain.

**Methods:**

Adults with EVD for ICH who were receiving antibiotics, including vancomycin, ceftazidime, cefepime and meropenem, for VAI from January 2023 to April 2024 in two north Taiwan medical centers were enrolled in this study. The primary outcome was the area under the concentration (AUC) ratio between CSF and plasma (AUC_CSF/plasma_). Factors influencing CSF concentration was also investigated. In addition, the probability of target attainment (PTA) of anti-Pseudomonal antibiotics were calculated.

A summary of the permeability of vancomycin into the cerebrospinal fluid in this study.

**Figure:**
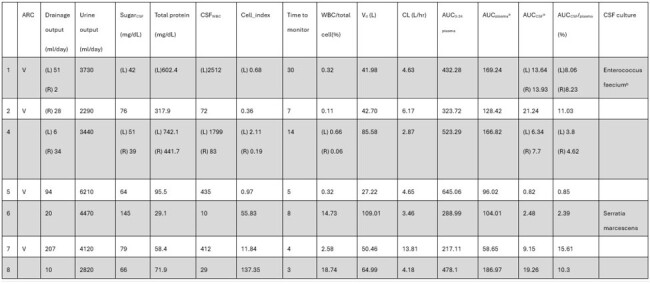

ARC augmentation renal clearance. L Left-side external ventricular drain R Right-side external ventricular drain.

a. AUC was calculated from the first observation point to the last observation point.

b. It was resistant to vancomycin.

**Results:**

A total of 9 patients (6 male) with VAI were enrolled, and contributed 7 vancomycin, 3 ceftazidime, 2 cefepime and 2 meropenem treatment courses. One patient provided two courses. The ranges of AUC_CSF/plasma_ for vancomycin, ceftazidime, cefepime, or meropenem were as follows 0.85-15.61%, 6.74-15.61%, 0.16-128.73%, and 0.09-13.24%, respectively. The CSF to plasma concentration ratio at the end of vancomycin infusion and at the middle of dosing interval showed good correlation with AUC_CSF/plasma_ (Spearman's coefficient 0.8 and 0.783, p-value ≦0.01) For ceftazidime, both CSF and plasma achieved at least 98% fT >MIC=8 under 2 gram every 8 hours regimen. For meropenem, the fT >MIC=2 for CSF and plasma were 0-100 % and 95.7-99.8% under 2 gram every 8 hours regimen, respectively. For cefepime, the fT >MIC=16 for CSF and plasma were 0-100% and >97%, respectively.

**Conclusion:**

This is the first study to report real-world data of antibiotic CSF penetration in neurosurgical population in Taiwan. Ceftazidime showed adequate penetration and achieved sufficient PTA in the CSF. Additionally, the single-point ratio of vancomycin at the end of infusion and during the mid-dosing interval may serve as a surrogate for AUC_CSF/plasma_.

**Disclosures:**

**All Authors**: No reported disclosures

